# Pain-related nociceptive evoked potential and skin wrinkle test in small fiber neuropathy

**DOI:** 10.1590/0004-282X-ANP-2021-0327

**Published:** 2022-04-20

**Authors:** Otto Jesus HERNÁNDEZ FUSTES, Cláudia Suemi Kamoi KAY, Paulo José LORENZONI, Renata Dal-Prá DUCCI, Michelle Zonkowski RIBAS, Lineu Cesar WERNECK, Rosana Herminia SCOLA

**Affiliations:** 1 Universidade Federal do Paraná, Complexo Hospital de Clínicas, Departamento de Clínica Médica, Serviço de Neurologia, Serviço de Doenças Neuromusculares e Desmielinizantes, Curitiba PR, Brazil.; 2 Universidade Federal do Paraná, Curitiba PR, Brazil.

**Figure 1.**
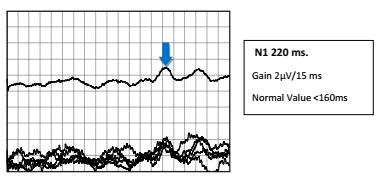
Pain-related nociceptive evoked potential.

**Figure 2.**
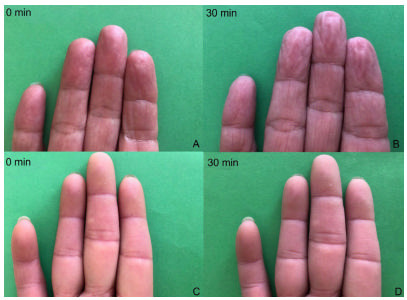
Skin wrinkle test. Patient (C and D). Normal test (A and B).

A 58-year-old woman with clinically probable small fiber neuropathy (SFN)^1^, with normal electroneuromyography, was evaluated with pain-related nociceptive evoked potential (PREP) with a concentric planar electrode stimulation on the dorsum of the hands and feet^2^ and skin wrinkle test (SWT)^3^. The PREP showed N1 latency delay (Figure 1), compatible with the change in SWT induced by water, which indicated a Grade 0 pattern (Figure 2), as can be seen in sympathetic autonomic small fiber dysfunction.

New electrophysiological tools, combined with SWT and specific neurological examination, contribute to the diagnosis of SFN.

Multiple-choice questions with related answers are given in Supplementary Material. The following material is available online for this article: https://www.arquivosdeneuropsiquiatria.org/wp-content/uploads/2022/02/ANP_2021327_material-suplementar_16-02.pdf
